# EGPA Phenotyping: Not Only ANCA, but Also Eosinophils

**DOI:** 10.3390/biomedicines11030776

**Published:** 2023-03-03

**Authors:** Andrea Matucci, Emanuele Vivarelli, Margherita Perlato, Valentina Mecheri, Matteo Accinno, Lorenzo Cosmi, Paola Parronchi, Oliviero Rossi, Alessandra Vultaggio

**Affiliations:** 1 Immunoallergology Unit, Careggi University Hospital, Largo Brambilla 3, 50134 Florence, Italy; 2 Immunology and Cellular Therapy Unit, Department of Experimental and Clinical Medicine, Careggi University Hospital, 50134 Florence, Italy; 3 Immunoallergology Unit, Department of Experimental and Clinical Medicine, Careggi University Hospital, 50134 Florence, Italy

**Keywords:** EGPA, eosinophils, vasculitis, phenotype, ANCA

## Abstract

Background: Eosinophilic Granulomatosis with Polyangiitis (EGPA) is a small-vessel necrotizing vasculitis. The anti-neutrophil cytoplasmic antibodies’ (ANCA) role in defining clinical EGPA phenotypes is well established. Although the role of eosinophils in disease pathogenesis has been clearly demonstrated, the value of blood eosinophil count (BEC) as a biomarker of disease phenotypes is currently uncertain. Methods: We retrospectively analyzed EGPA patients referred to our Immunology Clinic. Demographic, laboratory and clinical features were retrieved from clinical records, and a Logistic Regression was fitted to evaluate the predictive power of all baseline clinical and laboratory features to define EGPA phenotypes. Results: 168 patients were recruited. BEC ≤ 1500 cells/mL was predictive of a clinical involvement characterized by asthma, chronic rhinosinusitis with nasal polyps (CRSwNP) and lung opacities (OR 0.18, 95% CI 0.07–0.43; respiratory-limited phenotype); BEC > 3500/mL was predictive of extrapulmonary organ involvement (OR 3.5, 95% CI 1.7–7.1; systemic phenotype). BEC was also predictive of peripheral nervous system (PNS) involvement, with a positive trend with increasing BEC (<1500/mL: OR 0.17, 95%CI, 0.06–0.47; >3500/mL: OR 2.8, 95% CI, 1.5–5.28). ANCA positivity was also predictive of extrapulmonary involvement (OR 4.7, 95% CI 1.9–11.99). Conclusions: according to BEC and irrespective of the ANCA status, two EGPA phenotypes could be identified, named systemic and respiratory-limited phenotypes, with different organ involvement and possibly different prognoses.

## 1. Introduction

Eosinophilic Granulomatosis with Polyangiitis (EGPA) represents one of the most important clinical conditions in which eosinophils play a role in the disease pathogenesis. EGPA belongs to the group of anti-neutrophil cytoplasmic antibodies (ANCA)-associated vasculitis (AAV) [1]. In fact, EGPA is a rare disease characterized by granulomatous and eosinophil-rich inflammation and systemic necrotizing vasculitis affecting small-to-medium-size vessels [2]. Characteristically, EGPA develops in three sequential phases: the allergic, the eosinophilic and the vasculitic phases [3] but represents a diagnostic challenge. Eosinophils are the predominant cell type involved in EGPA inflammation; in fact, circulating and tissue eosinophilia are hallmarks of the disease and result from a deranged Th2 response [4]. From a pathogenetic point of view, in addition to eosinophils, the potential role of T cell subsets, such as Th2, Th17, and B cells has been suggested [5,6].

In the American College of Rheumatology (ACR) criteria, EGPA was defined by the presence or a history of asthma, a blood eosinophil percentage higher than 10% or an absolute count >1.000 cells/mm^3^ and the presence of at least two of the following features: (1) histopathological evidence of eosinophilic vasculitis or perivascular eosinophilic infiltration or eosinophil-rich granulomatous inflammation; (2) pulmonary opacities, non-fixed; (3) sino-nasal disease such as chronic rhinosinusitis with nasal polyps (CRSwNP); (4) neuropathy, characterized by mono- or poly-motor deficit or nerve conduction abnormality [7]. Recently, the ACR/European Alliance of Associations for Rheumatology has proposed new classification criteria for EGPA. These criteria confirm the prominent role of blood eosinophilia and asthma in distinguishing EGPA from other forms of vasculitis [8]. The relative importance of EGPA derives from its clinical complexity and epidemiological impact, particularly in asthmatic patients [9]. Asthma, characterized by an elevated blood eosinophil count (BEC), is a typical manifestation of EGPA and often presents several years before the disease onset, in association with CRSwNP. Therefore, EGPA could represent a possible evolution of eosinophilic forms of severe asthma [10]. Moreover, in asthmatic patients, an elevated BEC has been proposed as a potential biomarker of disease severity. In fact, it has been demonstrated that a higher BEC is associated with more severe exacerbations and poorer asthma control [11].

Interleukin- 5 (IL-5), mainly produced by T cells, but also IL-3 and granulocyte macrophage colony stimulating factor (GM-CSF), can activate eosinophils, prolong their survival and induce the expression on the cell surface of receptors and integrins necessary for tissue migration [4]. Tissue migration is a complex process mainly driven by chemokines, such as eotaxins-3 (CCL26), RANTES (regulated upon activation, normal T cells expressed and secreted, CCL5), monocyte chemoattractant protein (MCP)-3 (CCL7) and MCP-4 (CCL13) [3,4,10]. 

ANCA targeting myeloperoxidase (MPO) are known to play a relevant role in EGPA pathogenesis, even though they are usually found in about 30–40% of patients [12]. ANCA are produced by autoreactive B cells and can prime neutrophils and monocytes leading to tissue injury by reactive oxygen species (ROS) and cytokines production [12]. Activated neutrophils can also produce neutrophil extracellular traps (NETs) which further damage the vascular endothelium and stimulate ANCA production, because they contain DNA and several proteins, including MPO [12].

Clinical and epidemiological data indicate that the EGPA phenotypes seem to be influenced by the ANCA status. It has been observed that ANCA-positive patients present a vasculitic pattern of the disease, with multiorgan involvement, leading to peripheral neuropathy, progressive glomerulonephritis, gastrointestinal manifestations, etc. On the other hand, the major eosinophil-driven complications, such as myocardiopathy, are most frequently found in ANCA-negative patients [13,14,15,16].

Eosinophils contain a large number of mediators capable of inducing tissue damage, including basic proteins [eosinophils cationic protein (ECP), eosinophil neurotoxin (EDN), major basic protein (MBP)] but also cytokines and chemokines that can amplify the inflammatory process [17]. The toxic properties of eosinophil-derived proteins have been highlighted by several in vitro and in vivo studies, especially on cardiomyocytes and nerve fibers [18,19,20]. Eosinophils also have the capacity to induce the production of fibrogenic cytokines, such as Transforming Growth Factor (TGF)-β, IL-1α and IL-1β. This biological activity is deemed important in producing tissue fibrosis, as demonstrated in endomyocardial fibrosis and airways remodeling in EGPA patients. Furthermore, eosinophils are also able to activate the endothelium, thus fostering the formation of a vascular pathology; the formation of eosinophil extracellular traps (EETs), a DNA-protein complex resulting from a modified apoptotic process, can promote further tissue inflammation and vascular thrombosis [21,22].

The main aim of our study was to identify an easily obtainable biomarker, able to define the different disease phenotypes. Specifically, we focused on the role of eosinophils in defining EGPA phenotypes. We analyzed a cohort of patients referred to our Immunology Clinic to further understand the role of eosinophils as a disease severity and prognostic biomarker.

## 2. Materials and Methods

### 2.1. Study Population

**Figure 1:**
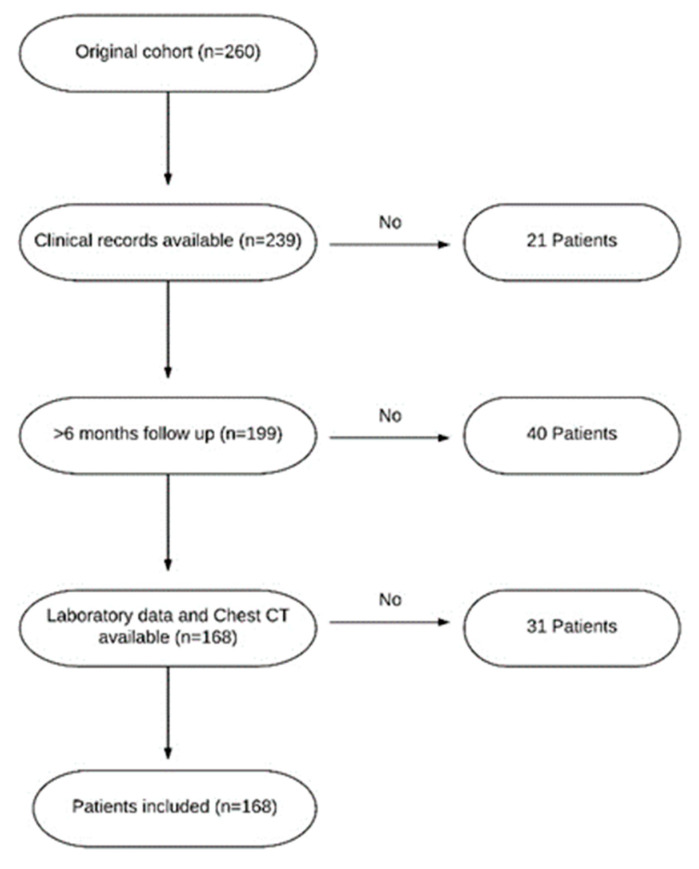
Inclusion and exclusion criteria for study enrollment. CT: chest tomography.

Among the patients referred to our Immunology Clinic from January 2010 to December 2021, patients fulfilling the 1990 ACR EGPA classification criteria were enrolled. Demographic, clinical and laboratory data were retrieved from clinical records; patients with incomplete clinical records or a short follow-up duration were excluded from our work (See Figure 1). Blood eosinophil count (BEC), total IgE (CAP-FEIA, ThermoFisher, Uppsala, Sweden) and ANCA (EUROPLUS Granulocyte Mosaic 25, IFA, Euroimmun, Lubeck, Germany; EliATM MPOS, EliaTM PR3S, ThermoFisher, Uppsala, Sweden) were analyzed at baseline and chosen as predictors for logistic regression. The atopic status definition was based on a positive clinical history and positive skin testing and/or specific IgE antibodies against common allergens. 

The preferential “respiratory-limited phenotype” was defined as the presence of lung involvement (lung infiltrates) without constitutional symptoms (persistent fever >38 °C, night sweats, unexplained weight loss) or skin, heart, joints, kidney and Central/Peripheral Nervous System (CNS/PNS) involvement. Conversely, the presence of constitutional symptoms and/or organ involvement, in addition to lung involvement, defined the “systemic phenotype” (lung plus extra-pulmonary disease). We did not include asthma and CRSwNP in the logistic regression model because they were present in all and almost all patients, respectively. Clinical involvement was assessed by the treating physicians at baseline and during the clinical follow-up. 

All patients were evaluated at baseline with: (1) nasal endoscopy and a head computed tomography (CT) scan (sino-nasal involvement); (2) chest CT scan (lung involvement); (3) transthoracic echocardiography (heart involvement); (4) serum creatinine analysis, urinalysis and kidney ultrasound (kidney involvement). The treating physicians also assessed PNS, skin and joints involvement during the first and each follow-up visits. More advanced assessments such as nerve conduction studies, cardiac magnetic resonance (MRI) or coronary angiography were performed according to the clinical needs.

### 2.2. Statistical Methodology

Statistical analysis was performed using Python 3.8.0 version (Anaconda distribution). The cohort’s descriptive variables are reported as mean ± standard deviation or proportions. A logistic regression model with several clinical variables, including BEC and ANCA, was applied to evaluate the variables’ predictive power for clinical involvement, using a *p* value lower than 0.05 to select the significant variables. Variables without significant predictive power were excluded from the final model. The chi square test was used to assess for differences in categorical variables; *p* values lower than 0.05 were considered statistically significant.

## 3. Results

### 3.1. Patients Characteristics and Clinical Phenotypes of EGPA

We recruited a population of 168 patients for our cohort study, mainly consisting of female patients (101/167; 60%), with a mean age at disease onset of 46.8 ± 13.6 years and a significant diagnostic delay from the beginning of EGPA symptoms of 3.2 ± 6.4 years. ANCA tested positive in more than one-third of the patients (64/168; 38.1%), and no significant difference in BEC was found between ANCA-positive and ANCA-negative patients. The mean serum IgE value was 505.7 ± 667 kU/L. Baseline BEC and blood eosinophil percentage were, respectively, 5416 ± 4988 cells/μL and 33.1 ± 15.3. 

Concerning the clinical EGPA characteristics, all patients had asthma, and CRSwNP was present in most of them (161/168; 95.8%). The small group of patients without CRSwNP was not different from the rest of the cohort in terms of sex, age at diagnosis, atopy and total IgE or other baseline features. Concerning the overall clinical characteristics, 45 out of the 168 patients (26.8%) displayed only lung opacities in addition to asthma and CRSwNP, whereas 123 patients (73.2%) showed, in addition to alveolar infiltrates, other organ involvement. Specifically, we observed mononeuritis/polyneuropathy in 53.6% of the patients (90/168), constitutional symptoms in 26.8% of the patients (45/168), skin rashes in 23.2% of the patients (39/168), arthritis in 20.2% of the patients (34/168), serositis in 10.7% of the patients (18/168), gut disease in 7.1% of the patients (12/168), cardiomyopathy in 6.5% of the patients (11/168) and glomerulonephritis in 5.9% of the patients (10/168). 

The clinical and laboratory features of the study cohort are summarized in Table 1.

### 3.2. Blood Eosinophils Count Is Predictive of Extra Lung Involvement

**Figure 2:**
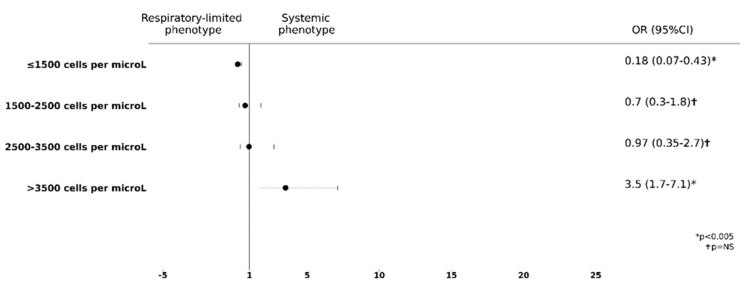
Prediction of extra-lung involvement using blood eosinophil count as a predictor (Logistic Regression).

To further analyze the role of eosinophils as potential biomarkers of disease pattern, we decided to evaluate the possible relationship between BEC and the risk to develop a preferential organ involvement in our cohort of EGPA patients. We divided our cohort of patients in four groups according to BEC: (a) ≤1500 cells/μL; (b) 1500–2500 cells/μL; (c) 2500–3500 cells/μL; (d) >3500 cells/μL. We observed that BEC ≤1500 cells/μL was significantly predictive of the “respiratory-limited phenotype” characterized by lung infiltrates in addition to asthma and CRSwNP [odds ratio (OR) 0.18 (95% CI 0.07–0.43)]. Conversely, an eosinophil count over 3500 cells/μL was predictive of the “systemic phenotype” which included, in addition to asthma and CRSwNP, at least one extra-pulmonary organ involved such as the PNS, kidney, heart, etc. [OR 3.5 (95% CI 1.7–7.1); see Figure 2]. In addition, patients with higher BEC levels also had a higher probability of testing positive for ANCA, although this result was without statistical significance (OR 1.78, CI95% 0.94–3.4, *p* = 0.08). 

### 3.3. Higher Blood Eosinophils Count Is Predictive of PNS Involvement

**Figure 3:**
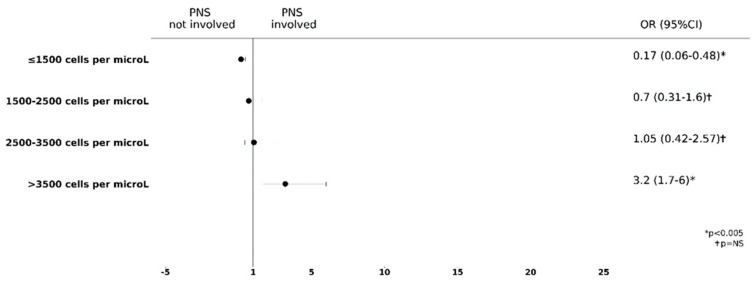
Prediction of PNS involvement using blood eosinophil count as a predictor (Logistic Regression).

In our previous work, we observed a positive association between BEC and PNS involvement in EGPA patients [23]. To gain a deeper insight in the value of eosinophils count as a predictive clinical biomarker in EGPA patients, we explored the impact of a progressive increase in baseline BEC on PNS involvement. Interestingly, we showed an increasing value of the OR for PNS involvement with progressively increasing BEC. Particularly, a BEC lower than 1500 cells/μL was associated with an OR of 0.17 (95%CI, 0.06–0.47), whereas, when BEC was higher than 3500 cells/μL, we observed a significant increase in the OR, reaching the value of 2.8 (95% CI, 1.5–5.28), clearly showing that the risk of PNS involvement was the highest in this subgroup of EGPA patients (Figure 3). More importantly, the predictive value of BEC for PNS damage was shown to be independent of the ANCA status, at least in our cohort. 

### 3.4. ANCA Positivity Is Predictive of a Preferential Extra-Pulmonary Organ Involvement

**Figure 4:**
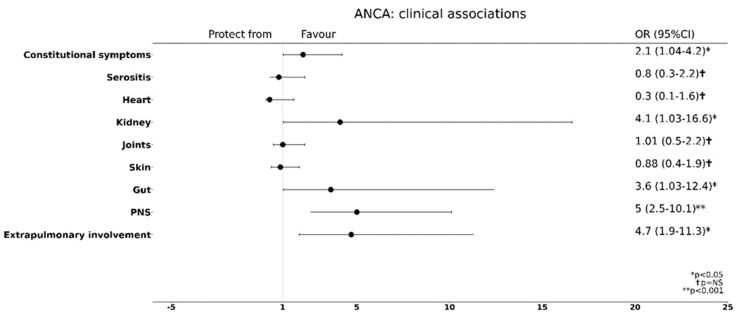
Prediction of organ involvement using ANCA as a predictor (Logistic Regression).

We then decided to evaluate the role of ANCA status as a biomarker of disease severity in terms of systemic involvement (systemic phenotype). Overall, any extra pulmonary organ involvement was more likely in patients who tested positive for ANCA [OR 4.7 (95% CI 1.9–11.99)]. Specifically, as shown in Figure 4, ANCA positivity, although demonstrated in a minor portion of patients (64/168; 38.1%), was strongly predictive of renal [OR 4.1 (95% CI 1.02–16.4)], gut [OR 3.5 (95% CI 1.02–12.3)] and PNS [OR 4.9 (95% CI 2.4–9.9] injury. Constitutional symptoms such as fever, weight loss, night sweats, etc. typically related to systemic vasculitis, also appeared to be predicted by ANCA positivity [OR 2.1 (95% CI 1.03–4.1)]. Conversely, in our case series, the presence of ANCA was predictive of heart involvement sparing, though this result did not reach statistical significance [OR 0.3 (95% CI 0.1–1.6)]. Joints and skin symptoms/signs were independent of the ANCA status. 

## 4. Discussion

In our study, we propose the existence of two main EGPA clinical phenotypes, based on the BEC but independent of the ANCA status, that we refer to as “respiratory-limited phenotype” and “systemic phenotype”. They are both characterized by upper and lower airways involvement with asthma and CRSwNP. The respiratory-limited phenotype is characterized by lung parenchymal involvement (eosinophilic alveolitis), whereas the second phenotype (systemic phenotype) is highly associated with extra-pulmonary disease. In particular, a BEC higher than 3500/μL is the predictive of systemic phenotype, although these results should be confirmed in larger cohorts. Further studies could refine this BEC cutoff. Traditionally, the EGPA clinical phenotypes, named “Inflammatory” and “Vasculitic” phenotypes, tend to be distinguished according to the ANCA status rather than to the percentage and/or number of eosinophils [13]. Concerning potential biomarkers useful to predict different clinical phenotypes of EGPA, it has been shown that PNS and renal involvement are more common in ANCA-positive patients, whereas cardiac involvement is typical of ANCA-negative patients [24]. The analysis of our cohort is in agreement with the literature data, showing the association of ANCA positivity with the systemic phenotype and, specifically, with the presence of gut, PNS, kidney involvement and constitutional symptoms. Although the presence of serum ANCA may be a useful biomarker of this clinical variant of EGPA, this cannot be considered an absolute criterion and, in any case, positivity for these autoantibodies can only be found in approximately 35–40% of patients, as in our case series [2,3]. Furthermore, the correlation between ANCA and EGPA is complex considering that the ANCA titers do not always correlate with disease severity and ANCA persist or reappear also in clinical remission phases [25].

Blood and tissue eosinophilia represents the cornerstone markers of all EGPA phases. Indeed, eosinophils are implicated not only in the first but also, by definition, in the second stage of EGPA, characterized by eosinophilic infiltration in tissues, as well as in the third overlapping phase of the disease in which vasculitis of the small arteries is associated with eosinophilic granulomas [12,26]. The pathogenic pathways of EGPA, representing the biological background underpinning the different forms of the disease, are considered to be eosinophilic inflammation on one side and systemic necrotizing vasculitis affecting small-to-medium-sized vessels on the other [27,28]. However, a pivotal observation is the fact that asthma and CRSwNP, two eosinophil-driven clinical manifestations, represent a hallmark of EGPA, detectable in all patients, even in those with the vasculitic variant. As for severe forms of type 2 asthma, nasal polyposis and asthma present in EGPA patients are sustained by an eosinophilic inflammation [29]. After all, EGPA is classically considered a Th2-driven inflammatory disease with a high eosinophil pattern in which an additional autoimmune response can also be present in a limited number of patients, as shown by ANCA production [4,29].

The peripheral blood eosinophil count is easily obtained, and its use as a diagnostic biomarker of EGPA is well accepted [7,8]. In our previous work, we observed that a high eosinophil count was associated with an increased risk of PNS involvement [23]. After all, Nishi and coworkers showed that the neurotoxic effect of eosinophils is related to the infiltration of these cells in the endoneurium and epineurium in patients with PNS involvement [30]. In addition, eosinophilic infiltrates and granulomas are found with a similar frequency both in ANCA-positive patients and in ANCA-negative patients, although, in ANCA-positive patients, histologically proven vasculitis is found more frequently and can cause tissue damage [13]. Eosinophils actively participate in the inflammatory process and tissue damage of the different organs involved, by releasing toxic mediators such as EDN, MBP, ECP and ROS (radical oxygen substances) and eosinophil extracellular DNA traps (EET) [31]. Overall, a central role for eosinophilic inflammation can be speculated regarding the extra-pulmonary involvement, both in ANCA-positive and in ANCA-negative patients.

**Figure 5:**
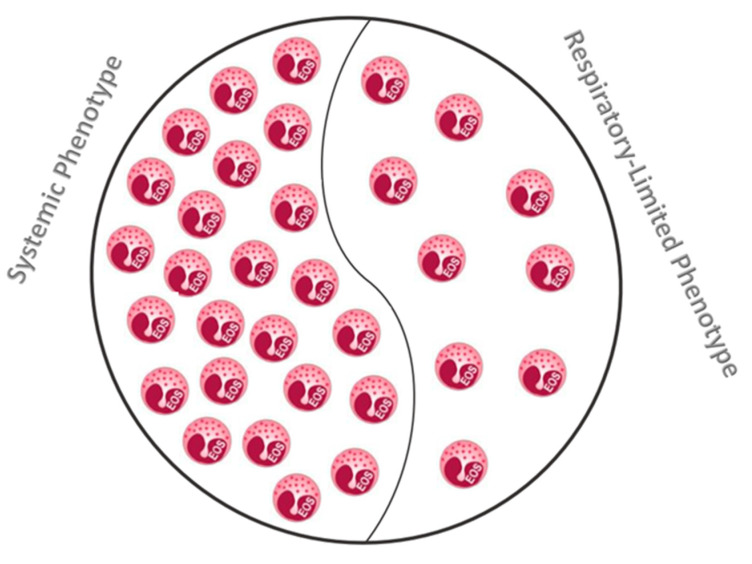
Schematic representation of the predictive value of eosinophils in distinguishing the respiratory-limited and systemic EGPA phenotypes.

A deep analysis of our study population suggested that a BEC of less than 1500 cells/μL was clearly associated with a preferential “respiratory-limited phenotype”, and this association tended to decrease significantly for higher values of eosinophils. Indeed, BEC values higher than 3500 cells/μL were significantly associated with the “systemic phenotype”, irrespective of the ANCA status. Therefore, the eosinophil count not only can be considered a key predictive marker of EGPA severity, but also could be used for phenotype definition (See Figure 5). The predominant role of eosinophilic inflammatory pathways may also suggest a potential theragnostic utility of BEC in predicting treatment responsiveness to biological agents targeting eosinophils, such as anti-IL-5 or IL-5α-receptor (IL-5R) monoclonal antibodies. After all, biological agents targeting eosinophils are able to induce a consistent reduction in the absolute eosinophil count, with concomitant clinical improvement in patients with eosinophilic disorders, such as severe eosinophilic asthma, CRSwNP and hypereosinophilic syndrome (HES) [32,33,34,35,36,37]. Preliminary studies have shown the efficacy of mepolizumab in the treatment of patients with EGPA [38], and the treatment with this mAb has been recently approved [39]. However, it must be underlined that patients with heart, kidney and nervous system involvement were excluded from the trial, thus the efficacy of this treatment has only been demonstrated for the forms that we defined as the “respiratory-limited phenotype” [39]. Moreover, we cannot exclude the possibility of efficacy of an anti-IL-5/IL-5R treatment also in patients with extra-pulmonary involvement, considering that eosinophils play a central pathogenetic role in the organ injury occurring in EGPA.

Although our analysis does not allow to define a clear dichotomy between the clinical forms of EGPA, BEC seems to be a useful marker of the main two forms of the disease. Overall, our study has several strengths. The cohort size was wide, and it was a monocentric study, allowing for a strong consistency in diagnostic and follow-up protocols. The main drawback is its retrospective nature, which can be prone to bias; a bias in disease severity, as our Centre is a tertiary care center, cannot be excluded in our cohort. Another drawback is the limited sample size. Moreover, further studies are needed to confirm the role of eosinophils as a predictive biomarker of EGPA severity and clinical phenotypes.

## 5. Conclusions

In summary, our work suggests the role of eosinophil count as a biomarker for EGPA phenotyping. In fact, differently from ANCA which are found in approximately one-third of patients, hyper-eosinophilia, a typical feature of the disease, can be detected in all patients. Moreover, the eosinophil count could help clinicians to stratify the EGPA patients according to disease severity and tailor follow-up and therapeutic strategies, as required in a modern precision medicine approach. A strong advantage of eosinophil count as a biomarker is the ease of detection and the low costs of the exam; in addition, the eosinophil count may be useful to assess EGPA exacerbations and treatment response. A major drawback of this biomarker could be that it can be easily influenced by some medical therapies such as oral corticosteroids (OCS). 

## 6. Future Perspectives

The role of eosinophil count as a biomarker of disease severity could be of paramount importance, allowing better therapeutic choices for the different disease phenotypes.

## Figures and Tables

**Table 1:** Baseline characteristics of the study population. Data are presented as mean ± standard deviation or number (%).

Demographic, Clinical and Laboratory Features	Study Cohort (*n* = 168)
Female/Male	101/67
Age at diagnosis (y)	50.1 ± 12.9
Age at symptoms onset (y)	46.8 ± 13.6
Diagnostic delay (y)	3.2 ± 6.4
Atopy	68 (40.5)
Asthma	168 (100)
CRSwNP	161 (95.8)
Arthralgia/Arthritis	34 (20.2)
CNS involvement	1 (0.5)
Constitutional symptoms	45 (26.8)
Gut involvement	12 (7.1)
Heart involvement	11 (6.5)
Kidney involvement	10 (5.9)
Lung opacities	144 (85.7)
PNS involvement	90 (53.6)
Serositis	18 (10.7)
Skin involvement	39 (23.2)
FEV1 (%)	69.5 ± 22
Blood eosinophils (cells/μL)	5416 ± 4988
Blood eosinophils (%)	33.1 ± 15.3
ECP (μg/L)	75.7 ± 98.2
Total serum IgE (kU/L)	505.7 ± 667
ANCA+	64 (38.1)

ANCA: Anti-neutrophil Cytoplasmic Antibodies; CNS: Central Nervous System; CRSwNP: Chronic Rhinosinusitis with Nasal Polyps; ECP: Eosinophil Cationic Protein; FEV1: Forced Expiratory Volume in the 1st second; PNS: Peripheral Nervous System.

## Data Availability

Data are available at a reasonable request from EV.
